# Supplementary material for: Prognostic Role of CSF β-amyloid 1–42/1–40 Ratio in Patients Affected by Amyotrophic Lateral Sclerosis
Source: Brain Sci. 2021 Feb 27;11(3):302. doi: 10.3390/brainsci11030302 (PMC7997395; doi:10.3390/brainsci11030302)
Supplement: Supplementary file 1 [file brainsci-11-00302-s001.pdf]

brain sciences

MDPI

Article

Prognostic Role of CSF β-amyloid 1–42/1–40 Ratio in Patients Affected by Amyotrophic Lateral Sclerosis

Tiziana Colletti [1], Luisa Agnello [2], Rossella Spataro [3], Lavinia Guccione [1], Antonietta Notaro [1], Bruna Lo Sasso [2], Valeria Blandino[4], Fabiola Graziano [4], Caterina Maria Gambino [2], Rosaria Vincenza Giglio [2], Giulia Bivona [2], Vincenzo La Bella [1], Marcello Ciaccio [2,†] and Tommaso Piccoli [4,†,*]

Supplementary Table 1. Neuropsychologic assessment of ALS patients by FrSBe, MMSE and ECAS at time of diagnosis (baseline).

Neuropsychologic Tests	Median	IQR
FrSBe	53	45.5-57.5
MMSE	27.7	24.3-29.5
ECAS	101	85-115

Supplementary Table 2. Multivariate Cox regression analysis for overall survival for ALS patients considering their co-morbilities.

Parameters	b	± SE	*p*	HR	95% C.I.
Smoke (yes *vs* no)	-0.090	0.410	0.826	0.914	0.409-2.041
Dermatological disorders (yes *vs* no)	-0.424	0.671	0.528	0.655	0.176-2.438
Respiratory disorders (yes *vs* no)	0.207	0.469	0.659	1.230	0.491-3.084
Otorhinolaryngological disorders (yes *vs* no)	0.260	0.576	0.652	1.297	0.419-4.015
Eye disorders (yes *vs* no)	0.610	0.595	0.306	1.840	0.573-5.904
Cardiovascular disorders (yes *vs* no)	0.623	0.319	0.05	1.865	0.999-3.483
Muscle-Skeletal disorders (yes *vs* no)	0.129	0.337	0.702	1.137	0.588-2.201
Gastroenteric disorders (yes *vs* no)	-0.220	0.313	0.492	0.803	0.434-1.484
Genitourinary disorders (yes *vs* no)	0.018	0.436	0.917	0.815	0.363-1.827
Dysmetabolic disorders (yes *vs* no)	-0.205	0.412	0.619	0.815	0.363-1.827
Psychic disorders (yes *vs* no)	-0.012	0.456	0.979	0.988	0.404-2-416
Hematologic disorders (yes *vs* no)	-0.22	0.524	0.675	0.803	0.288-2.240
Aβ 42/40 ratio	-22.001	8.889	**0.013***	2.79E-10	0.000-0.010
